# A Novel Mutation of the *HNF1B* Gene Associated With Hypoplastic Glomerulocystic Kidney Disease and Neonatal Renal Failure: A Case Report and Mutation Update

**DOI:** 10.1097/MD.0000000000000469

**Published:** 2015-02-20

**Authors:** Maria Inês Alvelos, Magda Rodrigues, Luísa Lobo, Ana Medeira, Ana Berta Sousa, Carla Simão, Manuel Carlos Lemos

**Affiliations:** From the CICS-UBI, Health Sciences Research Centre, Faculty of Health Sciences, University of Beira Interior, Covilhã, Portugal (MIA, MCL); Paediatric Nephrology & Renal Transplantation Unit, Department of Paediatrics, Santa Maria Hospital, Lisbon, Portugal (MR, CS); Department of Radiology, Santa Maria Hospital, Lisbon, Portugal (LL); and Genetics Unit, Department of Paediatrics, Santa Maria Hospital, Lisbon, Portugal (AM, ABS).

## Abstract

Hepatocyte nuclear factor 1 beta (*HNF1B*) plays an important role in embryonic development, namely in the kidney, pancreas, liver, genital tract, and gut. Heterozygous germline mutations of *HNF1B* are associated with the renal cysts and diabetes syndrome (RCAD). Affected individuals may present a variety of renal developmental abnormalities and/or maturity-onset diabetes of the young (MODY).

A Portuguese 19-month-old male infant was evaluated due to hypoplastic glomerulocystic kidney disease and renal dysfunction diagnosed in the neonatal period that progressed to stage 5 chronic renal disease during the first year of life. His mother was diagnosed with a solitary hypoplastic microcystic left kidney at age 20, with stage 2 chronic renal disease established at age 35, and presented bicornuate uterus, pancreatic atrophy, and gestational diabetes. DNA sequence analysis of *HNF1B* revealed a novel germline frameshift insertion (c.110_111insC or c.110dupC) in both the child and the mother. A review of the literature revealed a total of 106 different *HNF1B* mutations, in 236 mutation-positive families, comprising gross deletions (34%), missense mutations (31%), frameshift deletions or insertions (15%), nonsense mutations (11%), and splice-site mutations (8%).

The study of this family with an unusual presentation of hypoplastic glomerulocystic kidney disease with neonatal renal dysfunction identified a previously unreported mutation of the *HNF1B* gene, thereby expanding the spectrum of known mutations associated with renal developmental disorders.

## Abstract 2

Supplemental Digital Content is available in the text

## INTRODUCTION

Germline heterozygous mutations in the hepatocyte nuclear factor 1 beta gene (*HNF1B,* also termed *TCF2*) cause the renal cysts and diabetes syndrome (RCAD, OMIM #137920). This autosomal dominant disorder is associated with a wide clinical spectrum that includes abnormal renal development leading to nondiabetic renal disease, dysfunction of pancreatic β-cells leading to diabetes mellitus, and abnormalities of the liver and genital tract.^1,2^ This disorder has a wide phenotypic spectrum, and affected individuals may present isolated renal disease, isolated diabetes (maturity-onset diabetes of the young, MODY), or both. Although the *HNF1B* gene was initially associated with MODY type 5 (MODY5) diabetes,^3^ renal involvement is more prevalent in *HNF1B* mutation carriers, particularly in pediatric cases.^4^ Renal manifestations of *HNF1B* mutations include hypoplastic glomerulocystic kidney disease, cystic renal dysplasia, solitary functioning kidney, horseshoe kidney, and oligomeganephronia.^5–7^ A recent study revealed *HNF1B* mutations in 9% of adult patients with chronic renal failure of unknown origin.^8^ In addition, some individuals present urogenital abnormalities that include bicornuate uterus, bilateral agenesis of vas deferens, large epididymal cysts, and asthenospermia.^6^

*HNF1B* is located on chromosome 17q12 and comprises nine coding exons. *HNF1B* is a member of the homeodomain-containing superfamily of genes and encodes a widely distributed Pit-1/Oct-1/Unc-86 (POU) transcription factor with a major role in endodermal development, which explains the multiorgan involvement in affected patients.^9^ The majority of mutations in *HNF1B* consist of gene deletions, thereby indicating that haploinsufficiency is likely to be the major molecular mechanism underlying this disorder.^10,11^

Genetic screening for *HNF1B* mutations in suspected cases represents an important tool for diagnosis, prognosis, treatment, and genetic counseling. Thus, the identification of an *HNF1B* mutation provides molecular confirmation of a clinical diagnosis, raises the possibility of coexisting malformations, which should be investigated, facilitates the correct choice of treatment (unlike some types of MODY, diabetes of *HNF1B* carriers is not sensitive to sulfonylurea medication, and early insulin therapy is required),^6^ and provides information about recurrence risks for patients and family members.

We report a novel *HNF1B* frameshift mutation in a Portuguese family with an unusual presentation of hypoplastic glomerulocystic kidney disease and neonatal renal disease, and present an update of all published mutations in this gene.

## CASE PRESENTATION

### Clinical Characterization

**FIGURE 1:**
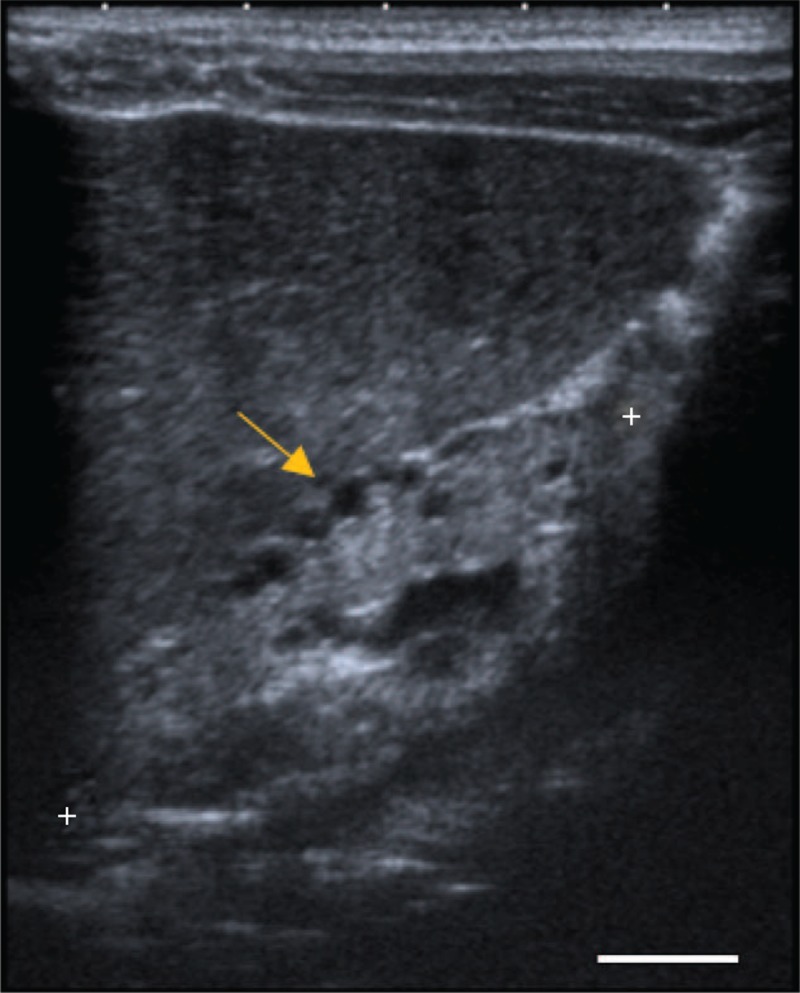
Longitudinal image of renal ultrasound scan performed at 12 months of age. The image shows a small-sized hyperechoic kidney, loss of corticomedullary differentiation, and multiple small cysts with a predominantly subcortical distribution (arrow), a feature that is highly suggestive of glomerulocystic renal disease. The kidney poles are represented as (+). A 10 - mm scale is represented by the horizontal bar.

**FIGURE 2:**
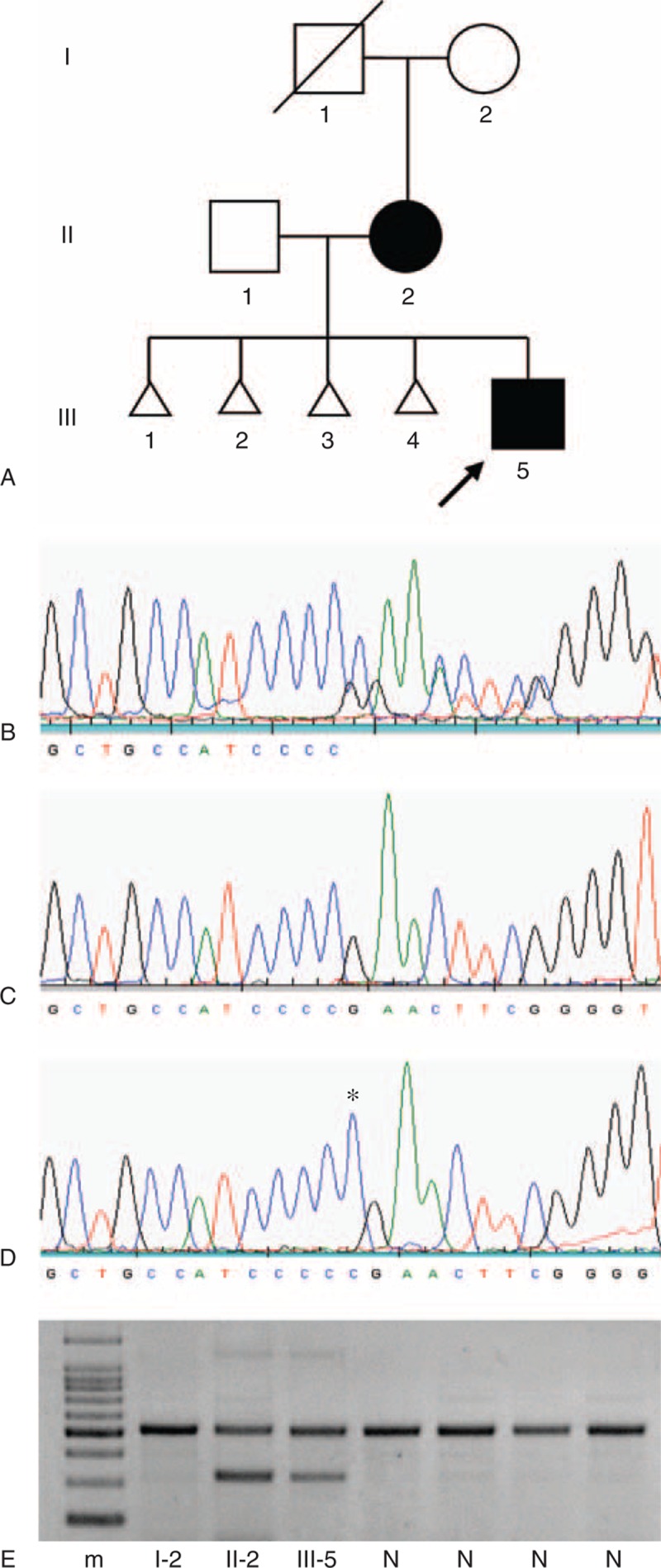
Identification of a germline frameshift insertion or duplication (c.110_111insC or c.110dupC) in the *HNF1B* gene in affected family members. (A) Pedigree of the family affected with hypoplastic glomerulocystic kidney disease, with the proband (III-5) indicated by an arrow. Individuals are represented as men (squares), women (circles), unaffected (open symbol), affected (filled symbol), deceased (oblique line through symbol), and miscarriages (triangles). (B) DNA sequence of the PCR product obtained from the proband, showing evidence of a heterozygous frameshift mutation. (C) DNA sequence of the normal allele, obtained through pGEM-T cloning of the PCR product from the proband. (D) DNA sequence of the mutated allele, obtained through pGEM-T cloning, showing the insertion (or duplication) of the additional cytosine (asterisk). (E) Agarose gel electrophoresis of a multiplex PCR using a 3’ modified forward primer complementary to the mutated allele. The affected individuals (II-2 and III-5) show a lower band (330 base pairs) corresponding to the amplification of the mutated allele, whereas this band is absent in the maternal grandmother (I-2) and in four normal controls (N). The upper band (529 base pairs) is an internal PCR control that results from amplification of exon 1. A 100 base-pair ladder molecular-weight marker (m) is shown. The mutation (c.110_111insC or c.110dupC) is numbered in relation to the *HNF1B* cDNA reference sequence (GenBank accession number NM_000458.2), whereby nucleotide +1 corresponds to the A of the ATG-translation initiation codon.

A 19-month-old male infant, first born child of nonconsanguineous Portuguese parents, was evaluated due to renal cysts and progressive renal disease diagnosed in the neonatal period. Pregnancy was complicated by maternal diabetes and chronic renal disease (CRD), and prenatal ultrasonography at 33 weeks’ gestation revealed hydramnios and large hyperechogenic kidneys. He was born by cesarean section performed at 35 weeks due to deteriorating maternal renal function. Apgar score at birth was normal, and weight and length were normal for gestational age. In the neonatal period, he was found to have elevated serum levels of creatinine, urea, and phosphorus, and a reduced glomerular filtration rate (GFR) (Table 1). Postnatal renal ultrasonography revealed slightly enlarged kidneys (longitudinal diameter: right 53 mm and left 51 mm), absence of corticomedullary differentiation, and diffuse hyperechogenicity with the presence of bilateral multiple small (≤5 mm) renal cysts with predominantly subcortical distribution. Careful evaluation did not identify any extra-renal malformations. The child was maintained under conservative therapy with nutritional management and dietary phosphate and potassium restriction, dietary phosphate binders, calcitriol, and folic acid, with dose adjustments according to blood chemistry results. During the first year of life, renal function impairment remained at stage 5 CRD (Table 1). At 1 year of age, renal ultrasonography showed kidney sizes smaller than expected for age (right 52 mm and left 55 mm), and with the same features as above (Figure 1). At 16 months of age, during an upper respiratory infection, renal function deteriorated, requiring initiation of substitutive therapy by peritoneal dialysis. No evidence for diabetes mellitus in the infant was found to date, as assessed by fasting plasma glucose and glycated hemoglobin (HbA1c). His mother, who was 34 years old at the time of birth, had been diagnosed with a solitary hypoplastic microcystic left kidney at age 20, with stage 2 CRD established at age 35. Additional investigations showed that she had extra-renal malformations, namely bicornuate uterus and atrophy of the body and tail of the pancreas. No history of diabetes mellitus was elicited, except for diet-treated gestational diabetes diagnosed at 25 weeks, with remission after delivery. She had a history of four previous miscarriages, but it was not possible to determine if the latter were due to her uterine abnormalities or corresponded to severely affected fetuses. There was no history of renal disease or diabetes in the maternal grandparents (Figure 2A).

**TABLE 1:**
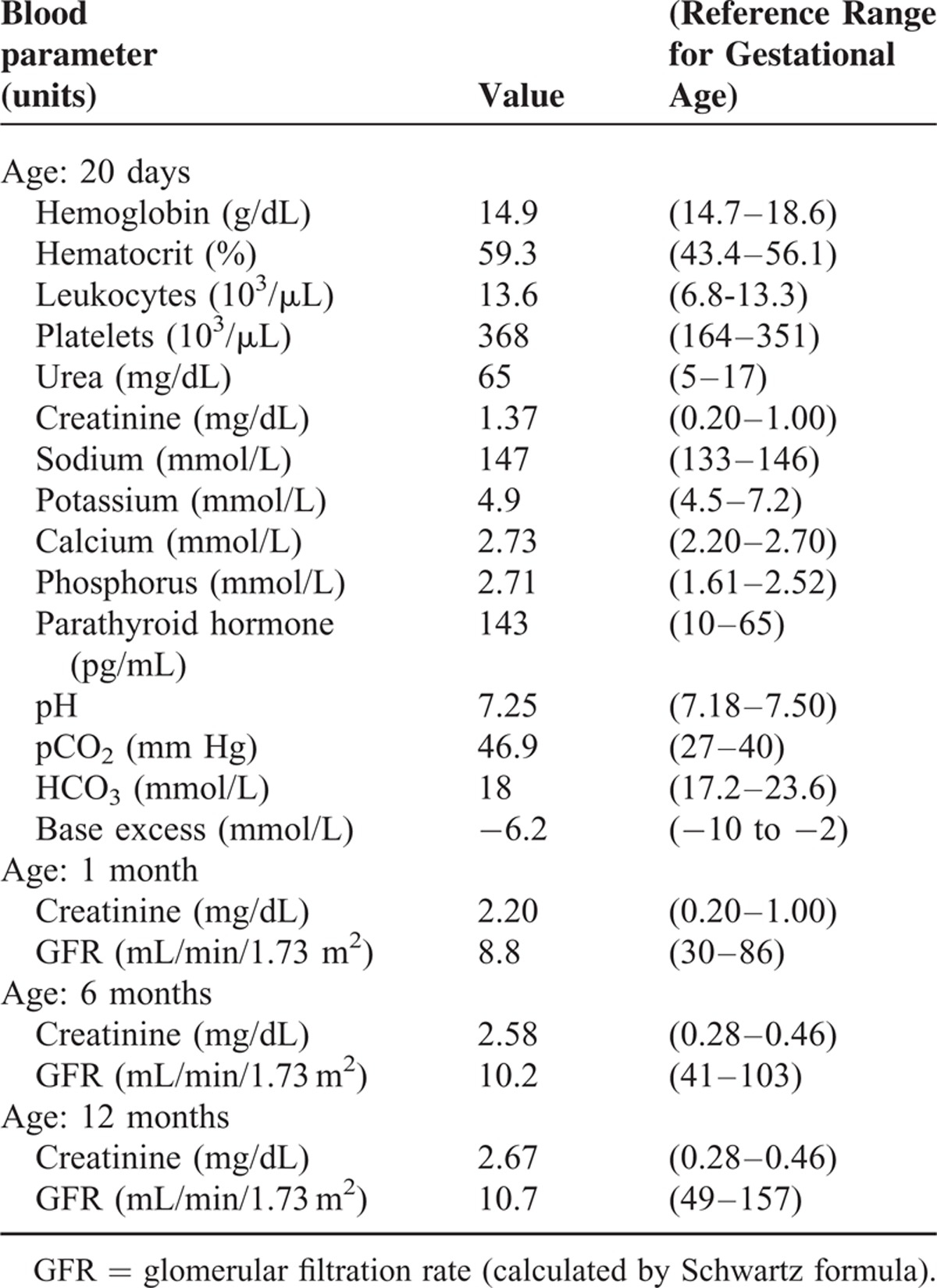
Laboratory Parameters in the Neonatal Period and Infancy

### Molecular Characterization

All genetic studies were approved by the Ethics Committee of the Faculty of Health Sciences, University of Beira Interior (Ref.: CE-FCS-2012-010), and written informed consent was obtained from all studied individuals or their legal guardian. Genetic screening of *HNF1B* in the affected child was performed using DNA extracted from peripheral blood leukocytes and polymerase chain reaction (PCR) amplification of all nine exons and exon-intron boundaries (primer sequences available upon request). Both strands were sequenced in forward and reverse direction using the CEQ DTCS (Beckman Coulter, Fullerton, CA, USA) sequencing kit following the manufacturer's recommendations, and analyzed on an automated capillary DNA sequencer (GenomeLab^TM^ GeXP, Genetic Analysis System; Beckman Coulter, Fullerton, CA, USA). PCR products were further analyzed by clone sequencing, using pGEM-T Easy Vector Systems (Promega Corporation, Madison, WI, USA). The molecular analysis of *HNF1B* revealed a heterozygous frameshift mutation in exon 1 (c.110_111insC, alternatively designated as c.110dupC) (Figure 2B–D), which is predicted to create a premature termination codon at position 87. The identification of this germline mutation led to the screening of other family members. The c.110_111insC mutation was present in the proband's mother (II-2), but not in his maternal grandmother (I-2) (Figure 2A). The presence of the mutation was also confirmed by an allele-specific multiplex PCR with *HNF1B* exon 1 primers (forward: 5’ GGGTGGAGGGGTTCCTGGAT 3’ and reverse: 5’ CGGGCGCAGTGTCACTCAGG 3’) and a mutation-specific primer with a 3’ additional C and a mismatched nucleotide (underlined) (forward: 5’ GAGTTGCTGCCAACCCCC 3’) that generated an amplicon only in the presence of the mutation (Figure 2E).

### Mutation Update

A list of published *HNF1B* germline mutations was obtained by searching the NCBI PubMed literature database for articles, using the keywords mutation combined with either *HNF1B* or *TCF2*. A total of 66 articles presented results of mutation analysis with at least one identified *HNF1B* germline mutation. A total of 106 different *HNF1B* mutations, in 236 mutation-positive families, were identified in the literature (Supplementary Table 1 http://links.lww.com/MD/A178). The distribution of mutation types in these affected families is gross deletions (34%), missense mutations (31%), frameshift deletions or insertions (15%), nonsense mutations (11%), and splice-site mutations (8%). Mutations are scattered across the gene, with no apparent hot spots, although they cluster predominantly in the first four exons, which encode the protein's binding domain. No strong genotype– phenotype correlation has been reported although there is some evidence that missense and frameshift mutations may be associated with a greater penetrance of diabetes and renal disease, respectively.^12^

## DISCUSSION

In the present study, we report a novel *HNF1B* mutation responsible for hypoplastic glomerulocystic kidney disease. This is also the first *HNF1B* mutation reported in a Portuguese family. In addition, this case report illustrates a remarkably variable expression of the disorder within the same family, with onset of renal failure ranging from the neonatal period (child) to adulthood (mother). This observation is consistent with previous reports of intrafamilial variability of the renal and nonrenal phenotypes, raising the possibility that additional genetic and/or environmental factors may modulate the expression of *HNF1B* mutations.^13,14^ Nevertheless, the neonatal onset of renal failure in the proband is quite atypical since the mean age of diagnosis in reported cases is approximately 21 years.^12^ The absence of diabetes in this family is not completely surprising as the reported prevalence of diabetes in mutation carriers is only about 45%, with a mean age of diagnosis of about 24 years, and in the great majority of cases, the diagnosis of diabetes occurs after the onset of the renal disease.^12^ It is, however, noteworthy that the mother developed gestational diabetes. The risk of diabetes exists and should be addressed by regular blood glucose monitoring.

The mutation identified in this family (c.110_111insC) consists of an insertion of a cytosine in exon 1 of one of the *HNF1B* alleles leading to a frameshift and a premature termination codon at position 87. The abnormal transcript may be degraded by a nonsense-mediated RNA decay mechanism, or may lead to a truncated nonfunctional protein due to the lack of specific domains such as the DNA-binding and transactivation domains, causing DNA binding impairment.^15^

The lack of clinical manifestations in both maternal grandparents and the absence of the mutation in the proband's grandmother (the deceased grandfather could not be tested) indicate that it is likely that a spontaneous *de novo* mutation occurred in the proband's mother. These spontaneous *de novo* mutations occur relatively frequently^16^; thus, testing for *HNF1B* mutations should not be discouraged by the absence of a family history of renal disease or diabetes.

In conclusion, our study of a family with an unusual presentation of hypoplastic glomerulocystic kidney disease with neonatal renal impairment identified a previously unreported mutation of the *HNF1B* gene, thereby expanding the spectrum of known mutations associated with renal developmental disorders.
